# 3D Imaging of Water-Drop Condensation on Hydrophobic and Hydrophilic Lubricant-Impregnated Surfaces

**DOI:** 10.1038/srep23687

**Published:** 2016-04-04

**Authors:** Tadashi Kajiya, Frank Schellenberger, Periklis Papadopoulos, Doris Vollmer, Hans-Jürgen Butt

**Affiliations:** 1 Max Planck Institute for Polymer Research, Ackermannweg 10, 55128 Mainz, Germany

## Abstract

Condensation of water from the atmosphere on a solid surface is an ubiquitous phenomenon in nature and has diverse technological applications, e.g. in heat and mass transfer. We investigated the condensation kinetics of water drops on a lubricant-impregnated surface, i.e., a micropillar array impregnated with a non-volatile ionic liquid. Growing and coalescing drops were imaged in 3D using a laser scanning confocal microscope equipped with a temperature and humidity control. Different stages of condensation can be discriminated. On a lubricant-impregnated hydrophobic micropillar array these are: (1) Nucleation on the lubricant surface. (2) Regular alignment of water drops between micropillars and formation of a three-phase contact line on a bottom of the substrate. (3) Deformation and bridging by coalescence which eventually leads to a detachment of the drops from the bottom substrate. The drop-substrate contact does not result in breakdown of the slippery behaviour. Contrary, on a lubricant-impregnated hydrophilic micropillar array, the condensed water drops replace the lubricant. Consequently, the surface loses its slippery property. Our results demonstrate that a Wenzel-like to Cassie transition, required to maintain the facile removal of condensed water drops, can be induced by well-chosen surface hydrophobicity.

Condensation is a core process for thermal management and heat transfer in power generation^1^,^2^. It also has a potential to supply water by dew or fog harvesting^3^,^4^,^5^,^6^ and desalination^7^,^8^,^9^. The enhancement of mass and heat transfer via condensation enables considerable savings in energy and natural resources^10^.

To design an optimal surface for continuous water condensation, two requirements must be satisfied: a high rate of drop nucleation and rapid removal of drops from the surface^11^,^12^,^13^. The latter requirement is crucial because each removed drop creates a new dry surface for fresh nucleation and prevents cloaking of the surface^14^,^15^. Therefore, drop-wise condensation on non-wettable surfaces is preferred over film-wise condensation on hydrophilic surfaces^16^.

To achieve efficient water-drop condensation, researchers have implemented diverse types of superhydrophobic surfaces. Superhydrophobic surfaces are patterned with hydrophobic micro- or nano-structures such that air is entrapped below a deposited water drop placed on top^17^,^18^,^19^,^20^. On a scale much larger than the surface patterning, such surfaces exhibit low contact angle hysteresis and enables a facile drop removal. The drops roll off tilted surfaces by gravity or by jumping off the surface when two drops coalesce^21^,^22^,^23^,^24^. However, facile drop removal is possible only when the interstitial space in the micro- and nano-structure maintains the air layer (Cassie state)^25^,^26^. The air layer acts as an isolation shield, lowering the heat transfer rate. Furthermore, water vapor may also condense inside the structure, resulting in water coverage of the micro-structured surface. (Wenzel state)^27^. The drops adhere tightly to the surface and the efficiency of drop removal is low.

An alternative approach is to use micro- and nano-textured surfaces impregnated with a non-volatile lubricant that is barely miscible with water^28^,^29^,^30^. On liquid-impregnated surfaces (LIS), the lubricant covers the solid surface because of the surface microstructure. Anaud *et al*.^31^ and Kim *et al*.^32^ have demonstrated that condensed drops can easily be removed on LIS by gravity. The LIS also decreases the energy barrier for drop nucleation, which can enhance the condensation rate^33^, because the water condenses on the deformable surface of the lubricant rather than on the hard solid substrate^34^,^35^.

Anaud *et al*. have also stressed the importance of the choice of lubricant^36^. The condensation efficiency is substantially influenced by the spreading coefficient S at the lubricant/water interface: S = *γ*_*l*_−*γ*_*w*_−*γ*_*wl*_, where *γ*_*l*_ is the surface tension of the lubricant, *γ*_*w*_ is the surface tension of water and *γ*_*wl*_ is the interfacial tension between water and the lubricant. For the condensed drop to grow without being cloaked by lubricant, the spreading coefficient must be negative.

Although LISs are promising for efficient water condensation, their application remains limited by insufficient understanding. The topology of the solid micro- and nano-structures and the presence of lubricant greatly influence the different stages of the condensation process^37^, i.e., the nucleation, growth, coalescence and removal of large drops. Previous analyses of drop growth and removal have been primarily based on the images recorded by video microscopy. However, video microscopy permits only the shape of the drop/air interface to be monitored. The drop/lubricant and drop/substrate interfaces remain hidden.

Here, we report the 3D observation of condensing water drops on a model LIS (a micropillar array impregnated with ionic liquid). To monitor the shape and position of micrometer-sized water drops *in situ*, optical imaging was performed using a laser scanning confocal microscope^38^,^39^,^40^ integrated with temperature and humidity control. The system permitted the drop/lubricant, lubricant/substrate and drop/substrate interfaces to be investigated with micrometer resolution. We describe the variations of the shape and position of each drop during condensation, and how they are influenced by parameters, such as the topology of the micropillars and the wetting property of the micropillars’ surface.

## Materials and Methods

### Materials

The SU-8 micropillar arrays were prepared via photolithography using SU-8 photoresist and a developer (Microchem, USA). After spin-coating the resist onto a glass slide, the substrate was baked at 95 °C for 4 min, cooled to 25 °C, exposed to UV light (mercury lamp at 350 W) for 35 s, and baked again at 95 °C for 4 min. Micropillars with two different geometries were prepared with rectangular and circular cross sections. The pillar width *w* and spacing *s* were 10, 20 and 50 μm at a fixed ratio *w*:*s* = 1 : 1 (*w* corresponds to the length of the side of the rectangular pillars and the diameter of the cylindrical pillars). The height of pillars was 10 μm.

The micropillars were treated by O_2_ plasma for 0.8 min at 150 W to clean the surface and to increase the density of OH groups. To prepare hydrophobic micropillar arrays, the pillars were, fluorinated with (1H,1H,2H,2H)-perfluorooctyl-trichlorosilane via chemical vapor deposition for 3 h prior to coating with lubricant. Alternatively, LISs with hydrophilic pillars were prepared by infiltrating the substrate with lubricant immediately after O_2_ plasma treatment.

Then, the micropillars were infiltrated with the ionic liquid 1-butyl-2,3-dimethylimidazolium bis(trifluoromethanesulonyl)imide. A drop of ionic liquid was deposited on the substrate. The substrate was left 1 hour for the ionic liquid drop to homogeneously infiltrate the structure. The height of the lubricant was adjusted by removing part of the ionic liquid with a tissue and waiting until the thickness became homogeneous, which was verified by confocal microscopy. The surface tension of water (saturated with ionic liquid) and ionic liquid (saturated with water) was measured using the pendant drop method (OCA35; DataPhysics, Germany), which yielded values of *γ*_*w*_ = 44 mN/m and *γ*_*l*_ = 35 mN/m, respectively. The water/IL interfacial tension was *γ*_*wl*_ = 11 mN/m. The measured value of the water surface tension in the presence of ionic liquid was lower than that of pure water, because a tiny amount of the ionic liquid was dissolved in water; the solubility of the ionic liquid in water was measured to be 0.97 ± 0.03 wt%. This system has a negative spreading coefficient *S*, which prevents cloaking of water drops after nucleation. The ionic liquid was fluorescently labeled with Lumogen Red F300 (BASF) F67/4. The hydrophobic dye did not show a notable surface activity.

To check the wetting property of the flat hydrophobic and hydrophilic SU-8 surfaces, the static contact angles of water (*θ*_*W*_) and ionic liquid (*θ*_*IL*_) drops were measured (OCA35; DataPhysics, Germany). The measured values were *θ*_*W*_ = 5 ± 2° and *θ*_IL_ = 16 ± 2° for hydrophilic SU-8 and *θ*_*W*_ = 110 ± 2° and *θ*_*IL*_ = 78 ± 1° for hydrophobized SU-8.

### Laser Scanning Confocal Microscope with Condensation Cell

**Figure 1:**
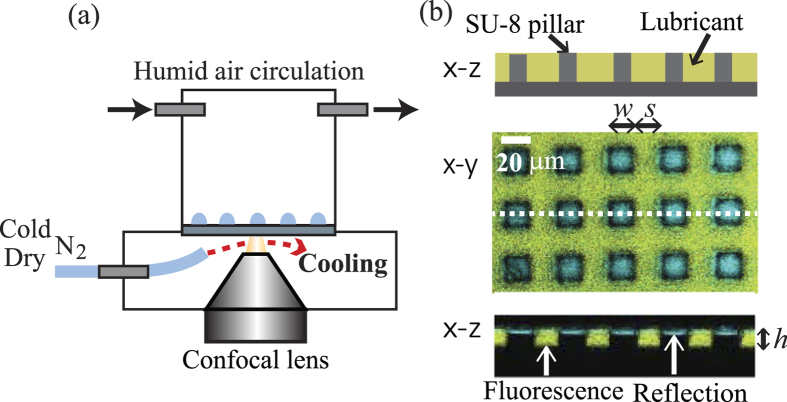
(**a**) Schematic representation of the temperature and humidity controlled measurement cell in the confocal microscope. (**b**) Schematic and confocal images of the LIS. For a confocal image, the *x−y* and *x−z* slices are shown. The yellow region corresponds to the ionic liquid. The blue color is the reflection from the interface. The pillars and substrate appear black. The pillar geometry is characterized by width (*w*), spacing (*s*) and height (*h*).

To simultaneously measure the shape of the condensing drops and the lubricant, a laboratory-built laser scanning confocal microscope with a blue laser (wave length: 473 nm, power: 25 mW, Cobolt, Sweden) and a 40 × /0.85 dry objective lens (Olympus, Japan) was used (Fig. 1a). A resonant scanner with a line frequency of 8 kHz (Cambridge Technology, USA) was used for the x−y scans. For the z-scan, the objective was moved by a piezo-stage (Nano F200, Mad City Labs, USA), leaving the sample at rest. This ensures that mechanical vibrations can be excluded, as it would affect drop growth and coalescence. The spatial resolution was approximately 400 nm in horizontal direction and 1 μm in vertical direction.

Eight times of line accumulations were applied to increase the signal to noise ratio. The acquisition of 3D images was processed at a frame rate of approximately 0.1 fps. The scanning range was 90 μm in length, 180 μm in width and 30 μm in height. Using two detectors with an optical filter, the emission and reflection of light were measured simultaneously.

To initiate water condensation on the LIS, a temperature control stage operated by gas cooling and a humidity control cell (cylindrical cell with diameter 50 mm and height 60 mm) were mounted on the confocal microscope. First, the LIS was put into the cell and was cooled down to a temperature of 4.0 ± 1.5 °C. The initial cooling process proceeded under constant circulation of dry air inside the cell (temperature and humidity: *T* = 20 ± 1.0 °C and *RH* = 10 ± 5%, flow rate: 250 ml/min. The values were checked by a sensor and flowmeter). To cool the lubricant-impregnated surface without condensing water on the back side of the substrate and the surface of the objective lens, cold dry nitrogen gas (*T* = 3.0 ± 1 °C, *RH* < 0.2% at gas outlet) was blown between the substrate and the objective lens. An IR camera confirmed that the spatial temperature variation on the LIS was less than 1 °C. After the LIS was cooled to the desired temperature, humid air (*T* = 20 ± 1.0 °C, *RH* = 80 ± 3%) was circulated in the cell to initiate condensation.

Figure 1b shows a *x−z* sketch as well as *x−y* and *x−z* images of the LIS substrate obtained using the confocal microscope. The image analysis was performed using ImageJ. The yellow color indicates the emission of light from the fluorescence molecules dispersed in the ionic liquid. The blue color represents the reflection of the incident light at interfaces with different optical indices. The micropillars appear black. The ionic liquid filled the space between the pillars (the filling height was 10 μm) but did not cover the top faces of the micropillars.

The filling height of ionic liquid remained unaltered during several successive repetitions of the condensation experiments. We did not observe an effect of water dissolution in ionic liquid. The nucleation density and the shape of the drops were identical within our experimental accuracy between successive measurements on the identical surface.

## Results and Discussion

### A. Drop condensation on hydrophobic micropillar arrays

**Figure 2:**
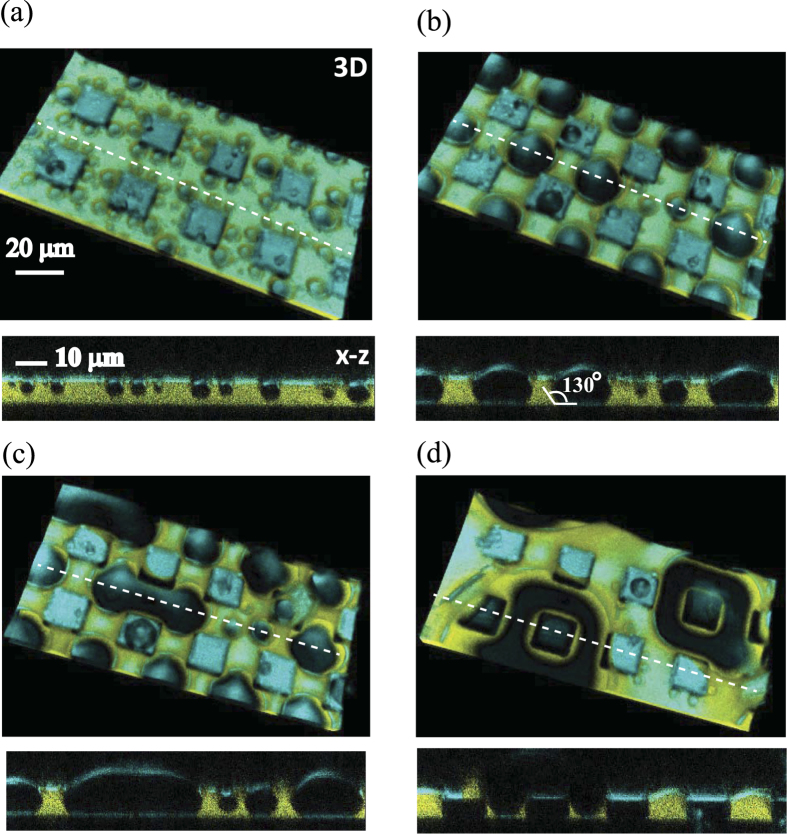
3D images and x−z cross-section of water drops condensing on an ionic liquid impregnated surface consisting of hydrophobic rectangular micropillars (*w* = 20 μm, *s* = 20 μm, *h* = 10 μm). Note that the 3D image is tilted for a clear visualization. In reality, the substrate is placed horizontally. Four sequential time steps are shown. (**a**) 70 s: Tiny droplets nucleate on the surface of the lubricant. (**b**) 300 s: As the size of the drops become comparable to the pillars’ spacing, they move between the pillars and formed a regular pattern. The drop contacts the bottom of the substrate with a contact angle of *θ* ≈ 130°. (**c**) 450 s: The drop deforms to fill the free space and bridge with its neighbor. (**d**) 600 s: The drops coalesce and form a large drop, the center of which covers a pillar. Movies of 3D images and x−y and x−z cross sections are available in the supplemental files S1 and S2.

An example for water drops condensing on the lubricant-impregnated hydrophobic rectangular micropillar arrays (*w*, *s* = 20 μm, *h* = 10 μm) is shown in Fig. 2. A movie of 3D images is available in supplemental information S1, and synchronized movies of x−y and x−z cross sections are available in S2. Drops grow in four stages:

- Nucleation (Fig. 2a): Tiny water drops (dark blue areas) nucleated mainly on the lubricant surface. As observed in the x−z cross section, the drops float on the lubricant surface and they are yet too small to get into contact with the substrate.
- Alignment (Fig. 2b): When the drop diameter (*d*) approaches the pillar spacing (*s* = 20 μm), the drop spontaneously moves to the largest area of the lubricant surface, i.e., the center of the square formed by the corners of four pillars. Consequently, the drops align regularly between the pillars. The *x−z* cross section reveals that the drops get into direct contact with the bottom substrate with a contact angle of *θ* = 132 ± 5°.
- Deformation (Fig. 2c): Due to the confinement of the pillars, the drop starts deforming to fill the space between the pillars. When neighboring drops touch, they bridge each other. As a result, the merged drops are elongated rather than forming spherical caps. The confinement by the micropillars delays coalescence.
- Coalescence (Fig. 2d): The coalescence of several drops forms a large spherical cap drop that covers the top faces of multiple pillars. The yellow region visible in the *x−z* cross section confirms that some lubricant remains in the corners between the bottom of the substrate and the wall of micropillars. These large drops move over large distances whereby they are coalescing, which leaves a fresh lubricant surface on which new water drops can nucleate.

In the subsequent sections, we provide a detailed discussions of the phenomena observed in the early (I-II) and late (III-IV) stages.

### B. Nucleation and growth kinetics at early stages I-II

On a LIS with hydrophobic pillars, most of the water drops nucleate and grow at the lubricant-air interface (Fig. 2a). This result is consistent with the theoretical analysis proposed by Eslami *et al*. and Anaud *et al*.^35^,^36^. The water-lubricant interface beneath the drop can deform to reduce the area of the high energy water-air interface^41^.

Drops nucleate and grow mostly near the edges of the micropillars. Nucleation is probably enhanced, because of the geometric discontinuities near edges^42^,^43^ and the curvature of the lubricant surface. Once drops have nucleated, they reduce the vapor pressure around them. This reduction is more efficient above the lubricant (because there are nucleated droplets) than on the top face of the pillars (since there are no droplets). The developing horizontal gradient in vapor pressure favors drops close to the edge to grow faster. The effect is enhanced by Ostwald ripening: Large drops grow at the expense of small drops because their vapor pressure, given by the Kelvin equation, is slightly lower. Furthermore, the smaller drops are absorbed by the larger drops via coalescence.

The drops floating on the lubricant move laterally. We attribute the lateral movement of the drops to a combined temperature and concentration-driven Marangoni effect. When a water drop condenses on the lubricant, the region around it heats up and creates a temperature gradient. Consequently, the surface tension of the lubricant surrounding the drop decreases. On a homogeneous surface, this heat is dissipated equally in all directions, but on the micropillar surface this symmetry is broken. Therefore, the surface tension on opposite sides of the drop may differ slightly, resulting in a Marangoni flow. In addition, a small amount of water is dissolved in the lubricant. This dissolution results in a change of the surface tension. Based on the same argument for temperature, we expect a Marangoni flow. The other possible mechanism is the capillary interaction by the lubricant meniscus^34^. As a meniscus of the lubricant is formed around the drop, it induces an attractive capillary interaction between neighboring drops. The phenomenon is similar to the Cheerios effect known for interaction between small particles floating on a liquid interface^44^,^45^. However, such interaction is short range and it does not play the main role for the lateral movement of drops. When the drop grows to a size of the pillar spacing, the drop stops moving as a result of the confinement due to the four pillars and the attachment with the bottom substrate.

**Figure 3:**
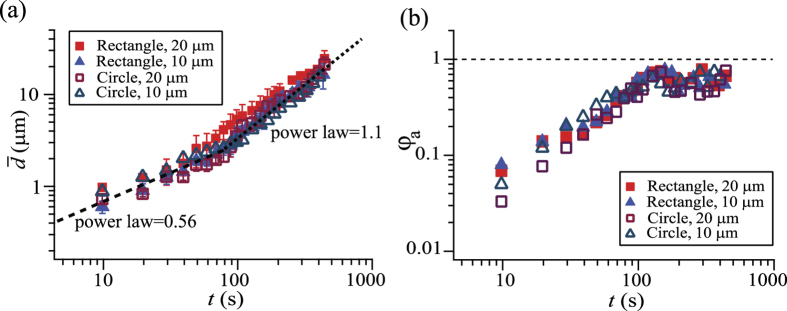
Averaged drop diameter 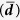 and area fraction of the surface occupied by condensed droplets (*φ*_*a*_) as a function of time. 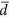
 and *φ*_*a*_ are measured at a height of the pillar’s top face (*z* = 10 μm). The pillars had a rectangular or circular cross-section of 10 μm or 20 μm width and identical spacing.

To quantify the growth of the water drops, we evaluated the number averaged drop diameter (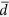) and area fraction (*φ*_*a*_) of the drops (Fig. 3). In the *x−y* cross section image sliced at the height of the top of pillars (*z* = 10 μm), we measured the area of the individual drops (*a*_*i*_, where *i* indicates the respective drops) and calculated the mean drop diameter (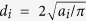). The number averaged drop diameter was obtained as follows:


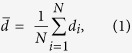


where *N* is the numbers of drops counted in the frame (*N* is typically 10 − 50 depending on the time *t*). The area fraction *φ*_*a*_ corresponds to the ratio of the surface area covered by the water drops compared to the total surface area of the lubricant (*A*_*l*_),


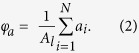


The pillars’ top faces are not considered in the calculation.

Two different growth laws can be distinguished. The drop grows in accordance with a power law with an exponent of 0.56 at the initial stage, independent on pillar shape and size. After approximately 100 s, the exponent changes to 1.1. The surface coverage *φ*_*a*_ monotonically increases during the first stage, and becomes nearly constant during the second stage. Such behaviour is analogous to that observed in condensation experiments on flat solid surfaces. In the first stage, the growth of individual drops is mainly due to vapor condensation on the drops’ surface^46^. In the second stage, the drop growth proceeds via the coalescence with other drops^10^. The observed exponents are close to the theoretical expectation, i.e., 1/2 for the initial diffusion-driven stage and 1 for the later coalescence-dominated stage.

Next, we discuss the shape of the drops (*x−z* cross sections in Fig. 2a,b). Because the spreading coefficient is negative, the top part of the drop is always surrounded by air. The drop shape is not spherical but like a lens. The air/water interface has a smaller curvature than the water/lubricant interface. As a result, the large portion of the drop penetrates into the lubricant.

**Figure 4:**
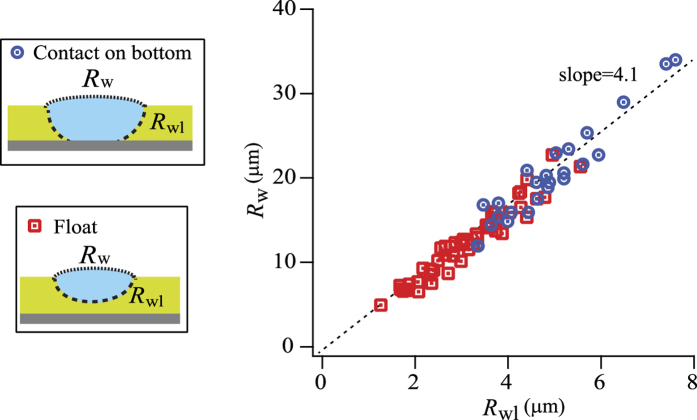
Radius of curvature of the drop at the air/water interface (*R*_*w*_) versus the radius of curvature at the water/lubricant interface (*R*_*wl*_). The red square symbols correspond to the drops floating on the lubricant and blue circular symbols correspond to the drops touching the bottom of the substrate. Data of different pillar sizes and geometries are superimposed in the graph. A linear fitting curve is plotted as dashed line with a slope of 4.1 ± 0.03.

In the sequential images of the *x−z* cross sections, we measured the radii of curvature at the air/water interface (*R*_*w*_) and the water/lubricant interface (*R*_*wl*_) (Fig. 4). The *R*_*w*_/*R*_*wl*_ ratio has a constant value of 4.1 ± 0.03, which remains constant even after the drop contacts the bottom of the micropillar arrays. The radii of curvature are determined by the ratio of the interfacial tensions^47^,^48^. Within the drop, the Laplace pressure is constant. The constant pressure implies that the interfacial tensions divided by the radii of curvature should be constant:


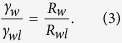


The *γ*_*w*_/*γ*_*wl*_ ratio was 44 (mN/m)/11 (mN/m) = 4, in excellent agreement with the experimental *R*_*w*_/*R*_*wl*_ result.

### C. Transition of wetting regimes at late stages III-IV

**Figure 5:**
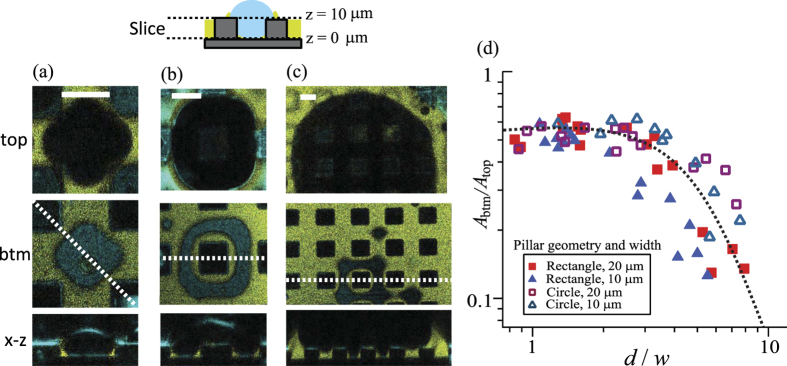
(**a–c**) Top and middle row: *x−y* cross section of a drop condensing on a LIS with hydrophobic rectangular micropillars. The images are sliced at the top (*z* = 10 μm) and bottom (*z* = 0 μm) surfaces of the pillars and recorded after (**a**) 200 s, (**b**) 550 s and (**c**) 1100 s. The white scale bar corresponds to 20 μm. Bottom row: images of the *x−z* cross section. As the drop size increases, the drop gradually detaches from the bottom surface. (**d**) Plot of the relative contact areas (*A*_*btm*_/*A*_*top*_) versus the ratio of the drop diameter and pillar width (*d*/*w)*. *A*_*btm*_ is the area of the bottom drop-substrate interface (z = 0 μm) and *A*_*top*_ is the area of the drop at the top of the pillars (z = 10 μm).

Figure 5a–c shows the *x−y* cross sections of the same drop at two different heights; one at the top of the micropillars with *z* = 10 μm and the other at the bottom of the micropillars with *z* = 0 μm. The *x−z* cross section at the white dashed line is also shown beneath.

As the drop size increases, the drop covers the top faces of a number of micropillars. At *t* = 200 s (Fig. 5a), the condensed water drop fills a large portion of the spacing between pillars on the top face of the lubricant (the coverage area is *A*_*top*_ ≈ 1.05 × 10^−10^ m^2^ at *z* = 10 μm). However, the contact area on the bottom of the substrate is considerably smaller (the contact area is *A*_*btm*_ ≈ 0.58 × 10^−10^ m^2^ at z = 0 μm). The ratio of the surface areas between these two heights is *A*_*btm*_/*A*_*top*_ = 0.55. Here, *A*_*btm*_ corresponds to the area of the drop surface in contact with the bottom of the substrate (*z* = 0 μm) and *A*_*top*_ indicates the area of the drop covering on the top of the pillars (*z* = 10 μm).

This tendency becomes more prominent at the later stage. At *t* = 500 s (Fig. 5b), the drop diameter increases to three times the pillar width and it completely covers a single micropillar. Meanwhile, the bottom contact area decreases compared to the top coverage area (*A*_*btm*_/*A*_*top*_ = 0.46). Notably, the bottom surface of the pillars is still surrounded by lubricant. The remaining lubricant meniscus around the bottom of pillars is stable because the lubricant forms its contact angle of 130° while still being able to maintain a water/lubricant interface with a curvature determined by the curvature of the drops air/water interface via Eq. 3.

When the drop diameter exceeds 5 or 6 times the pillar width and spacing (*t* = 1100 s, Fig. 5c), only a small fraction of the drop remains in contact with the bottom of the substrate. The main portion of the drop lifts towards the top faces of the micropillars.

To quantify how the drops reduce their contact area with the bottom surface, we plot a relative contact area (*A*_*btm*_/*A*_*top*_) as a function of the drop diameter scaled by the pillar width (*d*/*w*). (Fig. 5d). Until *d* ≈ 4*w*, *A*_*btm*_/*A*_*top*_ remains constant and independent of the geometry and size of the pillars. For *d* > 4*w*, the fraction of the drop in contact with the bottom of the substrate continuously decreases. The decrease in the contact area depends on the pillars’ spacing rather than the pillars’ geometry. Smaller pillar spacing favors complete lifting of the drop.

The observed detachment of the drop from the bottom surface is similar to the transition between the Cassie and Wenzel states observed on superhydrophobic micropillar surfaces^49^. The transition proceeds from the Wenzel-like state to the Cassie state^50^. The lifting of the drop minimizes the interfacial energy in two ways: by reducing the curvature of the droplet/lubricant interface that is confined by the micropillars and by reducing the contact area on the bottom and side wall of the pillars.

**Figure 6:**
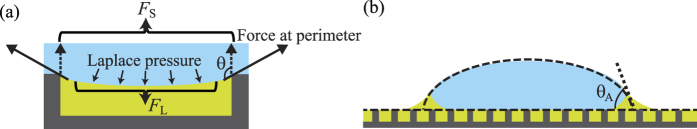
(**a**) Two forces acting around the pillar. The Laplace forces (*F*_*L*_) at the water/lubricant interface which pushes the drop downward and the force applied at the pillar perimeter that sustains the drop on the top (*F*_*S*_). *θ* is the contact angle at lubricant/water/solid boundary. (**b**) The apparent contact angle (*θ*_*A*_), determined as the angle of the intersection at which the contour of the air/water interface crosses the horizontal plane at a height of the pillars’ top faces.

Based on the studies for the wetting transition on superhydrophobic surfaces^51^,^52^, we estimate the critical condition for lifting condensed drops. The criteria for lifting follows from balancing the forces acting on the drop (Fig. 6a). The Laplace forces (*F*_*L*_) which pushes the drop downward and the force applied at the pillar’s perimeter that sustains the drop on the top (*F*_*S*_). Supposing that rectangular pillars of width (*w*) are aligned regularly with spacing (*s*), the *F*_*L*_ and *F*_*S*_ forces around single pillar can be estimated as follows:


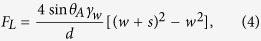



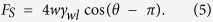


Here, *d* is the drop diameter, *θ* is the contact angle at lubricant/water/solid boundary, and *θ*_*A*_ is the apparent contact angle. The apparent contact angle (*θ*_*A*_) is determined as the angle of the intersection at which the contour of the air/water interface crosses the horizontal plane at a height of the pillars’ top faces (Fig. 6b).

The pillar width is equals to the spacing (*w* = *s*). Therefore, the critical drop diameter for the transition from the Wenzel-like state to the Cassie state is estimated as follows:


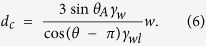


By substituting the experimental values (*γ*_*w*_ = 44 mN/m, *γ*_*wl*_ = 11 mN/m, *θ*_*A*_ = 40° and *θ* = 132°), *d*_*c*_ is estimated as 9 *w*. This result agrees with our experimental result (Fig. 5 (d)), *A*_*btm*_/*A*_*top*_  = 0 for *d*_*c*_ = 10*w*.

The transition from the Wenzel-like state to the Cassie state is facilitated by the coalescence of drops. When the drop coalesces with neighboring drops, the gain in surface energy is partially converted to kinetic energy, which agitates the drop-lubricant interfaces. In addition, the lubricant remaining around the bottom corner of pillars eases the detachment of drops.

### D. Drop condensation on hydrophilic micropillar arrays

Having the results obtained with hydrophobic micropillars, we now discuss the effect of the wetting property on the micropillars. Figure 6 shows 3D images and the *x−z* cross sections of water drops condensing on LIS prepared with hydrophilic micropillars. Movies are available in the supplemental information S3 (3D) and S4 (x−y and x−z cross sections). Although the pillar size and geometry are the same as those shown in Fig. 2, the condensation process is remarkably different from that on the LIS with hydrophobic micropillars.

**Figure 7:**
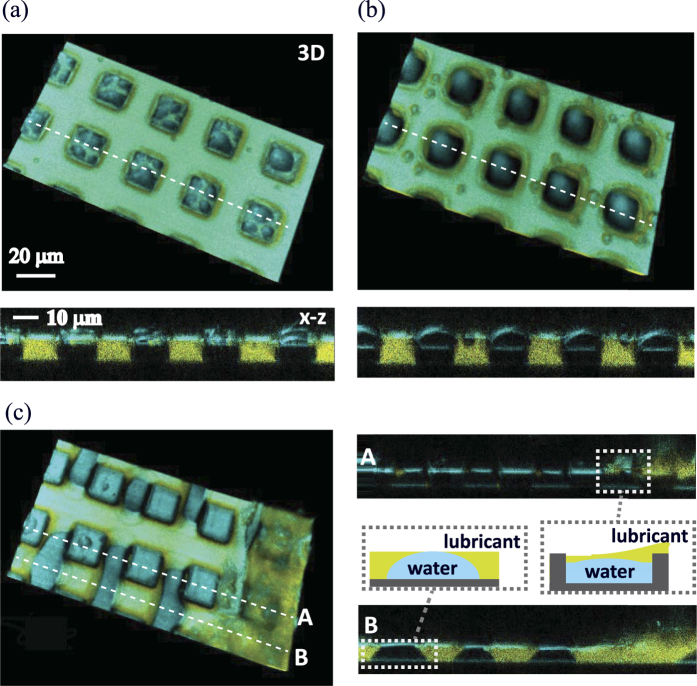
3D images and *x−z* cross sections of water drops condensing on a LIS composed of hydrophilic rectangular pillars (*w*, *s* = 20 μm, *h* = 10 μm). Images are recorded after (**a**) 50 s, (**b**) 200 s and (c) 260 s, respectively. The drops nucleate and grow preferentially on the pillar top surface. Few drops nucleate on a lubricant surface. In the later stage (**c**) the drops contact the pillars’ bottom surface. The water drops wet the bottom surface by displacing the lubricant, and the drops do not detach again. Movies of 3D images and x−y and x−z cross sections are available in the supplemental files S3 and S4.

The drops nucleate on the lubricant and the top surfaces the micropillars, with a preference for nucleation on the top surfaces of micropillars. Whereas the drops nucleating on the lubricant are mobile, the drops nucleating on the pillars’ top surface do not move (Fig. 7a,b). When these water drops grow and their bottom surfaces contact the substrate, the water drops wet the bottom and side of the micropillars, which displaces the lubricant (Fig. 7c). Finally, the lubricant is completely replaced by water, and the water strongly adheres on the micropillar surface. The LIS is no longer slippery.

**Figure 8:**
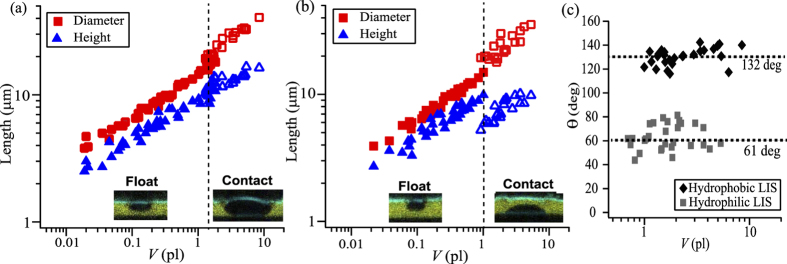
Plot of the diameter (*d*) and height (*h*) of the drop as a function of volume (*V*). The LISs with (**a**) hydrophobic and (**b**) hydrophilic micropillars are compared. Data for the different pillar sizes and geometries are superimposed in the graphs. (**c**) Apparent contact angle after the drop contacts the bottom solid surface.

To compare the drop growth process on the LIS with hydrophobic and hydrophilic micropillars, we measured the diameter (*d*), height (*h*) and volume (*V*) of individual condensing drops which nucleate and grow on the surface of the lubricant. Here, *d* was taken as the Feret diameter^53^ across the horizontal axis. In Fig. 8a,b, *d* and *h* are plotted as a function of *V* for the LISs with (a) hydrophobic and (b) hydrophilic pillars. For LIS with hydrophilic pillars, micropillars with large pillar spacing were used (w, s = 40 and 100 μm) in order to condense the water drop on the lubricant surface that is sufficiently apart from the top faces of hydrophilic pillars.

For small drops, *V* < 1 pl, no remarkable dependence on the pillars’ wettability was observed. The lense-shaped drop is floating on the surface of the lubricant by maintaining a nearly constant *d*/*h* aspect ratio. As the height approaches the thickness of lubricant layer and the drop contacts the bottom of the micropillars, the shape of the drop depends on the wetting property of the pillars. On the LIS with hydrophobic pillars, the drop has small drop/solid contact area and t and the diameter and height continuously increase with increasing drop volume. The *d*/*h* ratio remains almost unchanged. On the LIS with hydrophilic pillars, the diameter and height pass a discontinuity as soon as the drop contacts the bottom surface. The drop’s height decreases by almost 3 μm while the diameter increases by approximately 5 μm. The *d*/*h* ratio substantially changes once the drop contacts the bottom of pillars. The drop wets on the bottom solid surface by displacing the lubricant layer.

Figure 8c shows the contact angle versus the drop’s volume measured at the lubricant/water/substrate boundary. For drops that contact the bottom solid surface, *θ* is measured as approximately 132° for hydrophobic micropillars and 61° for hydrophilic micropillars. The contact angle remains constant and independent of the drop size.

The results demonstrate that the wetting property of micropillars is important for maintaining the slipperiness of the surface during the condensation process. When the micropillar surface is wettable with water instead of lubricant (LIS with hydrophilic pillars: *θ* = 61°), the growing drops spread in the spacing between pillars and the lubricant is displaced. Finally, most of the micropillar surface is covered by a water layer and the LIS loses its slippery property. On the other hand, if the micropillar surface is non-wettable with water (LIS with hydrophobic pillars: *θ* = 132°), the contact between the drop and the bottom solid surface is small, and a large portion of the solid surface remains surrounded by the lubricant. When these drops grow larger than the pillars’ spacing, the drops float on top of the pillars (Fig. 5). The LIS therefore maintains its slipperiness.

With a new 3D imaging of condensed water drops and their interfacial profiles, we have demonstrated clear evidence that on lubricant-impregnated surfaces, the transition from the Wenzel-like to Cassie states maintains the facile removal of condensed water drops. Our results also imply the importance of tuning the solid surface wettability before impregnation to realize the wetting transition.

In conclusion: Using confocal microscopy, we successfully imaged water drops condensing on lubricant-impregnated surfaces. Not only the drop surfaces, but also the drop/lubricant interface can be probed in 3D. Our results suggest the importance of tuning the solid surface wettability before impregnation. On lubricant-impregnated hydrophobic micropillars, water drops nucleate and grow on the lubricant surface. Once they have reached a size that their lower side contact the bottom substrate, the lubricant/water interface forms a define contact angle with the substrate. In this stage the drop’s mobility is restricted. When further growing at a diameter given by Eq. (6) the drops, now spanning several micropillars, detach again from the bottom substrate. On lubricant-impregnated hydrophilic micropillars, once the water drops contact the bottom substrate, they do not detach again. Their mobility is permanently restricted.

## Additional Information

**How to cite this article**: Kajiya, T. *et al*. 3D Imaging of Water-Drop Condensation on Hydrophobic and Hydrophilic Lubricant-Impregnated Surfaces. *Sci. Rep*. **6**, 23687; doi: 10.1038/srep23687 (2016).

## Supplementary Material

Supplementary Information

Supplementary Video S1

Supplementary Video S2

Supplementary Video S3

Supplementary Video S4

## References

[b1] Beer J. M. High Efficiency Electric Power Generation: The Environmental Role. Prog. Energy Combust. Sci. 33, 107–134 (2012).

[b2] Udell K. S. Heat Transfer in Porous Media Considering Phase Change and Capillarity – The heat pipe effect. Int. J. Heat Mass Transfer 28, 485–495 (1985).

[b3] Parker A. R. & Lawrence C. R. Water Capture by a Desert Beetle. Nature 414, 33–34 (2001). 11689930 10.1038/35102108

[b4] Zhai L. . Patterned Superhydrophobic Surfaces: Toward a Synthetic Mimic of the Namib Desert Beetle. Nano Lett. 6, 1213–1217 (2006). 16771582 10.1021/nl060644q

[b5] Park K. C., Chhatre S. S., Srinivasan S., Cohen R. E. & McKinley G. H. Optimal Design of Permeable Fiber Network Structures for Fog Harvesting. Langmuir 29, 13269–13277 (2013). 23895249 10.1021/la402409f

[b6] Rivera J. D. D. Aerodynamic Collection Efficiency of Fog Water Collectors. Atmos. Res. 102, 335–342 (2011).

[b7] Khawaji A. D., Kutubkhanah I. K. & Wie J. M. Advances in Seawater Desalination Technologies. Desalination 221, 47–69 (2008).

[b8] Alkhudhiri A., Darwish N. & Hilal N. Membrane Distillation: A Comprehensive Review. Desalination 287, 2–18 (2012).

[b9] Camacho L. M. . Advances in Membrane Distillation for Water Desalination and Purification Applications. Water 5, 94–196 (2013).

[b10] Beysens D. Dew Nucleation and growth. C. R. Phys. 7, 1082–1100 (2006).

[b11] Varanasi K. K., Hsu M., Bhate N., Yang W. & Deng T. Spatial Control in the Heterogeneous Nucleation of Water. Appl. Phys. Lett. 95, 094101 (2009).

[b12] Ghosh A., Beaini S., Zhang B. J., Ganguly R. & Megaridis C. M. Enhancing Dropwise Condensation through Bioinspired Wettability Patterning. Langmuir 30, 13103–13115 (2014). 25295388 10.1021/la5028866

[b13] Thickett S., Neto C. & Harris A. Biomimetic Surface Coatings for Atmospheric Water Capture Prepared by Dewetting of Polymer Films. Adv. Mat. 23, 3718–3722 (2011). 10.1002/adma.201100290 21766344

[b14] Miljkovic N. & Wang E. N. Condensation Heat Transfer on Superhydrophobic Surfaces. MRS Bulletin 38, 397–406 (2013).

[b15] Miljkovic N., Enright R. & Wang E. N. Modeling and Optimization of Superhydrophobic Condensation. J. Heat. Mass Trans. 135, 111004 (2013).

[b16] Schmidt E., Schurig W. & Sellschopp W. Kondensation von Wasserdampf in Film und Tropfenform. Technische Mechanik und Thermodynamik 1, 53–63 (1930).

[b17] Enright R., Miljkovic N., Obeidi A. A., Thompson C. V. & Wang E. N. Condensation on Superhydrophobic Surfaces: The Role of Local Energy Barriers and Structure Length Scale. Langmuir 28, 14424–14432 (2012). 22931378 10.1021/la302599n

[b18] Hou Y., Yu M., Chen X., Wang Z. & Yao S. Recurrent Filmwise and Dropwise Condensation on a Beetle Mimetic Surface. ACS Nano 9, 71–81 (2015). 25482594 10.1021/nn505716b

[b19] Rykaczewski K. . Multimode Multidrop Serial Coalescence Effects during Condensation on Hierarchical Superhydrophobic Surfaces. Langmuir 29, 881–891 (2013). 23259731 10.1021/la304264g

[b20] Zamuruyev K. O. . Continuous Droplet Removal upon Dropwise Condensation of Humid Air on a Hydrophobic Micropatterned Surface. Langmuir 30, 10133–10142 (2014). 25073014 10.1021/la5004462 PMC4148149

[b21] Wier K. A. & McCarthy T. J. Condensation on Ultrahydrophobic Surfaces and Its Effect on Droplet Mobility: Ultrahydrophobic Surfaces are not Always Water Repellent. Langmuir 22, 2433–2436 (2006). 16519435 10.1021/la0525877

[b22] Dorrer C. & Rühe J. Condensation and Wetting Transition on Microstructured Ultrahydrophobic Surfaces. Langmuir 23, 3820–3824 (2007). 17311432 10.1021/la063130f

[b23] Boreyko J. B. & Chen C. H. Self-Propelled Dropwise Condensate on Superhydrophobic Surfaces. Phys. Rev. Lett. 103, 184501 (2009). 19905808 10.1103/PhysRevLett.103.184501

[b24] Miljkovic N. . Jumping Droplet-Enhanced Condensation on Scalable Superhydrophobic Nanostructured Surfaces. Nano Lett. 13, 179–187 (2013). 23190055 10.1021/nl303835d

[b25] Extrand C. W. Designing for Optimum Liquid Repellency. Langmuir 22, 1711–1714 (2006). 16460095 10.1021/la052540l

[b26] Murakami D., Jinnai H. & Takahara A. Wetting Transition from the Cassie Baxter State to the Wenzel State on Textured Polymer Surfaces. Langmuir 30, 2061–2067 (2014). 24494786 10.1021/la4049067

[b27] Varanasi K. K., Deng T., Smith J. D., Hsu M. & Bhate N. Frost Formation and Ice Adhesion on Superhydrophobic Surfaces. Appl. Phys. Lett. 97, 234102 (2010).

[b28] Lafuma A. & Quéré D. Slippery Pre-Suffused Surfaces. Europhys. Lett. 96, 56001 (2011).

[b29] Wong T. S. . Bioinspired Self-Repairing Slippery Surfaces with Pressure-Stable Omniphobicity. Nature 477, 443–447 (2011). 21938066 10.1038/nature10447

[b30] Smith J. D. . Droplet Mobility on Lubricant-Impregnated Surfaces. Soft Matter 9, 1772–1780 (2013).

[b31] Anand S., Paxson A. T., Dhiman R., Smith J. D. & Varanasi K. K. Enhanced Condensation on Lubricant Impregnated Nanotextured Surfaces. ACS Nano 6, 10122–10129 (2012). 23030619 10.1021/nn303867y

[b32] Kim P. . Liquid-Infused Nanostructured Surfaces with Extreme Anti-Ice and Anti-Frost Performance. ACS Nano 6, 6569–6577 (2012). 22680067 10.1021/nn302310q

[b33] Xiao R., Miljkovic N., Enright R. & Wang E. N. Immersion Condensation on Oil-Infused Heterogeneous Surfaces for Enhanced Heat Transfer. Sci. Rep. 3, 1988 (2013). 23759735 10.1038/srep01988 PMC3680863

[b34] Steyer A., Guenoun P. & Beysens D. Hexatic and Fat-Fractal Structures for Water Droplets Condensing on Oil. Phys. Rev. E: Stat., Nonlinear, Soft Matter Phys. 48, 428–431 (1993). 10.1103/physreve.48.428 9960604

[b35] Eslami F. & Elliott J. A. W. Thermodynamic Investigation of the Barrier for Heterogeneous Nucleation on a Fluid Surface in Comparison with a Rigid Surface. J. Phys. Chem. B 115, 10646–10653 (2011). 21736344 10.1021/jp202018e

[b36] Anand S., Rykaczewski K., Subramanyam S. B., Beysens D. & Varanasi K. K. How Droplets Nucleate and Grow on Liquids and Liquid Impregnated Surfaces. Soft Matter 11, 69–80 (2015). 25410939 10.1039/c4sm01424c

[b37] Beysens D. & Knobler C. M. Growth of Breath Figures. Phys. Rev. Lett. 57, 1433 (1986). 10033448 10.1103/PhysRevLett.57.1433

[b38] Camara R. P. . Solid-Supported Thin Elastomer Films Deformed by Microdrops. Soft Matter 5, 3611–3617 (2009).

[b39] Papadopoulos P. . Wetting on the Microscale: Shape of a Liquid Drop on a Microstructured Surface at Different Length Scales. Langmuir 28, 8392–8398 (2012). 22578130 10.1021/la300379u

[b40] Schellenberger F. . Direct Observation of Drops on Slippery Lubricant-Infused Surfaces. Soft Matter 11, 7617–7626 (2015). 26291621 10.1039/c5sm01809a

[b41] Sokuler M. . The Softer the Better: Fast Condensation on Soft Surfaces. Langmuir 26, 1544–1547 (2010). 19928793 10.1021/la903996j

[b42] Medici M. G., Mongruel A., Royon L. & Beysens D. Edge Effects on Water Droplet Condensation. Phys. Rev. E: Stat., Nonlinear, Soft Matter Phys. 90, 062403 (2014). 10.1103/PhysRevE.90.062403 25615108

[b43] McCormick J. L. & Westwater J. W. Nucleation Sites for Dropwise Condensation. Chem. Eng. Sci. 20, 1021–1036 (1965).

[b44] Vella D. & Mahadevan L. The “Cheerios Effect”. Am. J. Phys. 73, 817−825 (2005).

[b45] Kralchevsky P. A. & Nagayama K. Capillary Interactions between Particles Bound to Interfaces, Liquid films and Biomembranes. Adv. Colloid Interface Sci. 85, 145–192 (2000). 10768480 10.1016/s0001-8686(99)00016-0

[b46] Sokuler M., Auernhammer G. K., Liu C. J., Bonaccurso E. & Butt H.-J. Dynamics of Condensation and Evaporation: Effect of Inter-Drop Spacing. Europhys. Lett. 89, 36004 (2010).

[b47] Knobler C. M. & Beysens D. Growth of Breath Figures on Fluid Surfaces. Europhys. Lett. 6, 707–712 (1988).

[b48] Jarvis T. J., Donohue M. D. & Katz J. L. Bubble Nucleation Mechanisms of Liquid Droplets Superheated in Other Liquids. J. Coll. Interface Sci. 50, 359–368 (1975).

[b49] Papadopoulos P., Mammen L., Deng X., Vollmer D. & Butt H.-J. How Superhydrophobicity Breaks Down. Proc. Natl. Acad. Sci. 110, 3254–3258 (2013). 23382197 10.1073/pnas.1218673110 PMC3587223

[b50] Rykaczewski K. . How nanorough is rough enough to make a surface superhydrophobic during water condensation? Soft Matter 8, 8786–8794 (2012).

[b51] Lafuma A. & Quéré D. Superhydrophobic States. Nature Mat. 2, 457–460 (2003). 10.1038/nmat924 12819775

[b52] Wang J. & Chen D. Criteria for Entrapped Gas under a Drop on an Ultrahydrophobic Surface. Langmuir 24, 10174–10180 (2008). 18698857 10.1021/la801092y

[b53] Merkus H. G. Particle Size Measurements: Fundamentals, Practice, Quality. Ch. 2, 14–15 (Springer 2009).

