# Supplementary material for: 3D Imaging of Water-Drop Condensation on Hydrophobic and Hydrophilic Lubricant-Impregnated Surfaces
Source: Sci Rep. 2016 Apr 4;6:23687. doi: 10.1038/srep23687 (PMC4819200; doi:10.1038/srep23687)
Supplement: Supplementary Information [file srep23687-s1.pdf]

3D Imaging of Water-Drop Condensation on Hydrophobic and Hydrophilic Lubricant-Impregnated Surfaces

Tadashi Kajiya, Frank Schellenberger, Periklis Papadopoulos†, Doris Vollmer and Hans-Jürgen Butt∗

Legends of the supplemental movie files S1-S4.

S1. 3D movie of water drops condensing on an ionic liquid impregnated surface consisting of hydrophobic rectangular micropillars (w = 20 µm, s = 20 µm, h = 10 µm). The movie proceeds 20 times faster than the real time. Note that the 3D image is tilted for a clear visualization. In reality, the substrate is placed horizontally.

S2. Movie of three cross sections obtained from the 3D data in S1. A x-y movie cross section at the height of the pillars' top faces (z = 10 µm), two x-z cross sections at the purple lines A and B. The movie proceeds 20 times faster than the real time.

S3. 3D movie of water drops condensing on an ionic liquid impregnated surface consisting of hydrophilic rectangular micropillars (w = 20 µm, s = 20 µm, h = 10 µm). The movie proceeds 20 times faster than the real time.

S4. Movie of three cross sections obtained from the 3D data in S3. A x-y cross section at the height of the pillars' top faces (z = 10 µm), two x-z cross sections sliced at the purple lines A and B. The movie proceeds 20 times faster than the real time.
